# Comparison of clinic-based assistance versus a centralized call center on patient-reported social needs: findings from a randomized pilot social health integration program

**DOI:** 10.1186/s12889-025-22334-x

**Published:** 2025-03-28

**Authors:** Ammarah Mahmud, Meagan C. Brown, Edwin S. Wong, India J. Ornelas, Robert Wellman, Roy Pardee, Sophia Mun, Ariel Singer, Emily Westbrook, Kathleen Barnes, Heidi Den Haan, Cara C. Lewis

**Affiliations:** 1 Department of Health Systems and Population Health, University of Washington, Seattle, WA USA; 2 Kaiser Permanente Washington Health Research Institute, Seattle, WA USA; 3 Department of Epidemiology, University of Washington, Seattle, WA USA

**Keywords:** Social health integration, Program evaluation, Social needs

## Abstract

**Background:**

As social need screening and intervention activities increase, the long-term objective of our work is to inform how to implement social health into healthcare settings. The purpose of this study is to assess changes in social needs over time between two social health support programs as part of a social health integration effort in two primary care clinics within an integrated health system in Washington state.

**Methods:**

We used stratified randomization to assign 535 patients who self-reported social needs on a screener between October 2022-January 2023 to one of two social health support programs: local, clinic-based Community Resource Specialists (CRS) or a centralized Connections Call Center (CCC). Participants were assessed at 2- and 5-months post-randomization. We compared the count of social needs across programs at each timepoint using joint tests, and estimated differences between programs using generalized linear mixed effects models at each timepoint.

**Results:**

We randomized 535 participants, with 270 assigned to CCC and 272 to CRS. Of those randomized, 61% completed at least one follow-up survey (*N* = 329). This analytic sample consisted of 153 CCC participants and 176 participants under CRS. CRS participants reported 0.08 (95% CI: -0.710, 0.864) more needs at 2 months and 0.42 (CI: -0.288, 1.126) more needs at 5 months compared to CCC participants (*p* > 0.05). An exploratory as-treated analysis within the CRS group suggested that referral receipt was associated with fewer needs over time.

**Conclusions:**

There were no significant differences between CRS and CCC participants’ social needs over time. However, receiving referrals to social services may lead to reduced social needs.

**Supplementary Information:**

The online version contains supplementary material available at 10.1186/s12889-025-22334-x.

## Introduction

Previous research estimates that social factors contribute to approximately 40% of individuals’ health status, compared to healthcare which accounts for about 20% of patients’ health status [1]. Individual-level social factors such as housing instability, food insecurity, and limited transportation manifest from social determinants of health and are associated with poor health outcomes, including cardiovascular disease, diabetes and poor diabetes management, as well as behavioral health conditions [2, 3]. When patients in healthcare settings request assistance with social factors, these are known as social needs [4]. Evidence from efforts to identify and respond to social needs in clinic and community settings, also known as social health integration, suggests that there is a positive association between receipt of social services and patient health outcomes [5–9].

As clinics increasingly engage in social health integration activities, the National Academies of Science, Engineering, and Medicine has provided guidance about five different types of integration activities: (1) awareness through identification of social risks, (2) adjustment by altering care that acknowledges patients’ social health, (3) assistance through resource connection, (4) alignment of health systems with community resources to address patient needs, and (5) advocacy by promoting policy change [10]. Clinics often prioritize “awareness” and “assistance” activities by using tools to screen for social needs and providing patients with resources and referrals to social service organizations, respectively [11]. Examples of interventions that link patients to resources can range from on-site community health workers to service call lines who help with resource navigation [11–13]. The Centers for Medicare and Medicaid Services (CMS) supported 28 sites to implement an Accountable Health Communities Model in which Medicare and Medicaid beneficiaries received screening, referrals, and navigation services to address social needs [14]. CMS’s recent framework for health equity also highlights the importance and need to collect patients’ social health data [15]. Professional and accreditation organizations have also emphasized the need to identify and address patients’ social health [16]. This aligns with recent payment policy reforms to incentivize these activities [17]. Most recently, the National Committee of Quality Assurance released a new Healthcare Effectiveness Data and Information Set quality measure for social need screening and intervention [18].

As these activities increase, it is essential to understand their effectiveness to further support patients. However, few studies have evaluated these types of programs and more rigorous studies are needed. Previous studies often also focused within individual clinical departments, or on a limited set of needs and subpopulations such as publicly insured beneficiaries, adults experiencing homelessness, or those with multiple chronic conditions [19–21]. Additionally, many studies have focused on intermediate process measures, such as the number of patients screened, rather than social needs or health outcomes [22].

There is also a need for greater research that includes more appropriate and stronger operationalization of measures. Previous studies have been inconsistent in how social needs and their resolution are operationalized as outcomes. For example, some have used the case manager responses or patient enrollment in resources as proxies for needs resolution [14, 23, 24]. This variability makes it difficult to synthesize findings and best practices across multiple studies. Additionally, most previous studies have used pre-post or cross-sectional study designs, leaving potential confounders or biases unaddressed [21, 25].

The purpose of our study was to examine the effects of a social health integration pilot program on patients’ social needs in a health care system. Kaiser Permanente (KP) is a national, not-for-profit integrated health care system and this program took place in the KP Washington region (KPWA) which provides medical coverage to about 700,000 members in Washington State. Under this program, participants in two primary care clinics could receive social health support from one of two interventions: a Community Resource Specialist (CRS) program, consisting of clinic-based health workers, or the Connections Call Center (CCC), a centralized call center program. The purpose of this study is to characterize and compare the presence of 10 social needs following the two interventions over time.

## Methods

KPWA identified two primary care clinics to receive implementation support for social health integration activities between July 2021– January 2023 as part of a quality improvement project. Examples of implementation support activities include virtual practice facilitation support to develop social health integration workflows and documentation to share with care teams, IT collaboration on tool design and training, and a monthly panel with patients with social needs to codesign integration workflows. Clinics were chosen based on their patient population diversity compared to other clinics in Washington State, and range in clinic size. They received implementation support to administer universal social health screening using a 9-item Social Health Questionnaire (SHQ). The SHQ consists of 8 items asking patients about the following social risks: food insecurity, housing insecurity, financial strain, and transportation issues. The final item asks patients if they desire assistance with up to 10 types of social needs. The SHQ is unique to KPWA and includes items that align with social risks that can be flagged in patients’ electronic health record (EHR). This instrument is available in English, Spanish, Russian, Mandarin, Korean, and Vietnamese. For patients younger than 18 years of age, the accompanying caregiver completed the SHQ on the patient’s behalf. Additional details about the development of the SHQ can be found in Additional file 1. Patients could complete their SHQ online in advance during electronic check-in, or in-person on paper or tablet at their visit. Responses were entered into patients’ EHR.

Patients who self-reported any social need on the last item of the SHQ during the enrollment period were assigned to one of two social health support programs: a local, clinic-based Community Resource Specialist (CRS) or a centralized national call center, Connections Call Center (CCC). Randomization occurred within strata based on clinic, age, and sex. We hypothesized that participants assigned to CRS would have a lower total count of social needs at each time point, indicating resolved social needs. We also reported the prevalence of all ten social needs by assigned social health support group at each time point. The KPWA Institutional Review Board determined that this was a quality improvement project and did not require Institutional Review Board review.

### Participants

Participants were included in the evaluation if they completed the SHQ during the enrollment period between October 2022 – January 2023, for a visit scheduled at one of the two pilot clinics, and reported any social need. Eligible visits included office visits with a primary care provider (excluding nurse and walk-in clinic visits) at primary care clinics (Family Practice, Pediatrics, and General Internal Medicine). They were excluded if they had a same-day referral to or encounter in the past month with a CRS, had a household member who was already randomized in the evaluation, spoke a language other than English or Spanish, or indicated opting-out of all outreach activities under the health system in their EHR.

The KPWA Research Institute Survey Research Program contacted participants to complete follow-up surveys at 2- and 5-months post-randomization. These surveys included SHQ items in addition to questions about their overall experiences. During the follow-up window, participants received an advance letter with a web link to the survey and a $2 pre-incentive, as well as an email or text message for the 5-month survey if they opted in to those contact methods at their 2-month survey. If the web version was not completed within a week, survey team members made 5 call attempts for participants to complete the survey over the phone. A paper survey was also sent to participants who did not respond to prior outreach attempts. A $25 incentive was provided for each survey that was completed. Our final analytic cohort included respondents who completed at least one follow-up survey, resulting in a final sample size of 329 participants (response rate = 61%).

### Social health support programs

Participants were assigned to receive social health support through one of two programs. CRSs are local, in-person specialists embedded in the care team and who reach out to participants to provide assistance [12]. CCC is a centralized call center administered by KP National. Both modalities provide participants with resource information for social needs and aim for the original agent to conduct follow-up. CRS and CCC staff have access to Thrive Local, a centralized resource database, and can share information from this platform with participants [26]. In addition to this, CRSs have local knowledge about community-based resources to which they can refer participants. CRSs are also embedded within care teams and receive training on motivational interviewing techniques to support participants’ social needs resolution.

CCC agents provide resources using Thrive Local and similarly initiate contact with participants. Prior to this evaluation, patients initiated contact to CCC to receive assistance. However, during a two-month period, we observed that less than 1% (2/214) of those who received CCC contact information in their after visit summary reached out to CCC. Given the quality improvement initiative driving this evaluation, we revised the CCC workflow so that the study team provided randomized participants’ contact information to CCC staff so they could initiate outreach.

### Outcomes

The primary outcome was the total count of social needs, ranging from 0-10, among participants randomized to the CRS program relative to CCC at each timepoint. We assessed this outcome using the SHQ item asking participants if they would like assistance and to select all that apply from the following social needs: food, housing, utilities, finances, transportation, loneliness or social isolation, employment, caregiving, childcare, or paying for medical care, medicine, or medical supplies.

### Predictors and covariates

The assigned social health support program was the main independent variable (CCC; CRS). Covariates included variables used for stratified randomization: administrative sex, age (< 18; 18–40; 41–60; 60 +), and clinic (A or B). We also included baseline count of social needs as a covariate. This aligns with best practices to include stratification variables and the baseline value for a continuous outcome as covariates in longitudinal, randomized studies [27, 28]. An analysis of the association between patient characteristics and outcome missingness led us to include insurance type (commercial; individual; Medicaid; Medicare; no coverage), race and ethnicity, and Johns Hopkins’ Adjusted Clinical Group (ACG) resource utilization band as covariates. ACGs are derived from patients’ previous utilization patterns using claims data, and categorizes patients into comorbidity levels based on their expected resource use [29]. We used administrative data instead of self-reported data from the survey for race and ethnicity due to high missingness for this item in the survey. We aggregated the race and ethnicity variable to five categories (African American/Black; Hispanic; Multiracial; White; Other; Unknown) due to small cell sizes. The “unknown” category includes individuals who refused or for whom the data was not collected. The “other” category consists of patients who selected “other” as their race and ethnicity, and those who identified as Native American or Alaska Native, Asian, or Native Hawaiian or Pacific Islander. We also used a binary ACG variable (No User/Low/Healthy; Moderate/High/Very High).

### Statistical analysis

We calculated descriptive statistics (proportions and standardized mean differences (SMDs)) for the total sample and by social health support programs, and identified meaningful differences between groups based on a SMD > 0.2 [30]. We estimated the difference in the count of social needs between CRS and CCC at each follow-up time point using a generalized linear mixed effects model assuming a Poisson distribution with log link and individual-level random effects for participants. We reported the unadjusted and adjusted mean counts of endorsed needs between programs for easier interpretation, in addition to risk ratios. Joint tests and associated *p*-values assessed if there was a statistically significant association at each timepoint between programs.

Primary analyses used an intent-to-treat approach. We conducted sensitivity analyses to account for potential self-selection bias as the analytic sample only included participants who responded to at least one follow-up survey [31]. Specifically, we used a two-part Heckman selection model which focused on who was included in the sample in the first part and used a main outcomes Poisson model as the second part (see Additional file 1, Appendix) [32]. This method produces unbiased estimates when working with missing data [33].

We also conducted an as-treated analysis using propensity score weights to measure the effect of receiving resources from CRS on the count of social needs compared to those who did not receive resources. Randomization did not guarantee that participants received assistance or used recommended services, and this secondary analysis focused on receipt of resources based on available data. We focused on CRS for the as-treated analysis because CRS is a unique, high touch program and we wanted to better understand effects among those who received this intervention. We defined receipt as those who spoke to a CRS and received resource information, and identified these participants by reviewing case notes. Our comparison group consisted of participants who did not receive any information from either CRS or CCC. We estimated a balanced comparison group using propensity score weights. Covariates used to create a comparison group included individual-level demographics and documented diagnoses for 26 comorbid conditions, clinic-level counts of primary care providers and full time CRSs, as well as a neighborhood-level deprivation index which measures regional socioeconomic conditions and is derived from geo-demographic data [34]. The sample size for those who received CRS was 104 and the comparison group consisted of 144 participants. Due to the small cell size of participants who received CCC, we could not create a balanced comparison group using propensity score weights and we were unable to conduct an as-treated analysis for CCC. Additional details about developing the as-treated dataset can be found in Additional file 1. We used Stata 17 and RStudio 4.2.1 for analyses.

## Results

### Participant characteristics

**Fig. 1:**
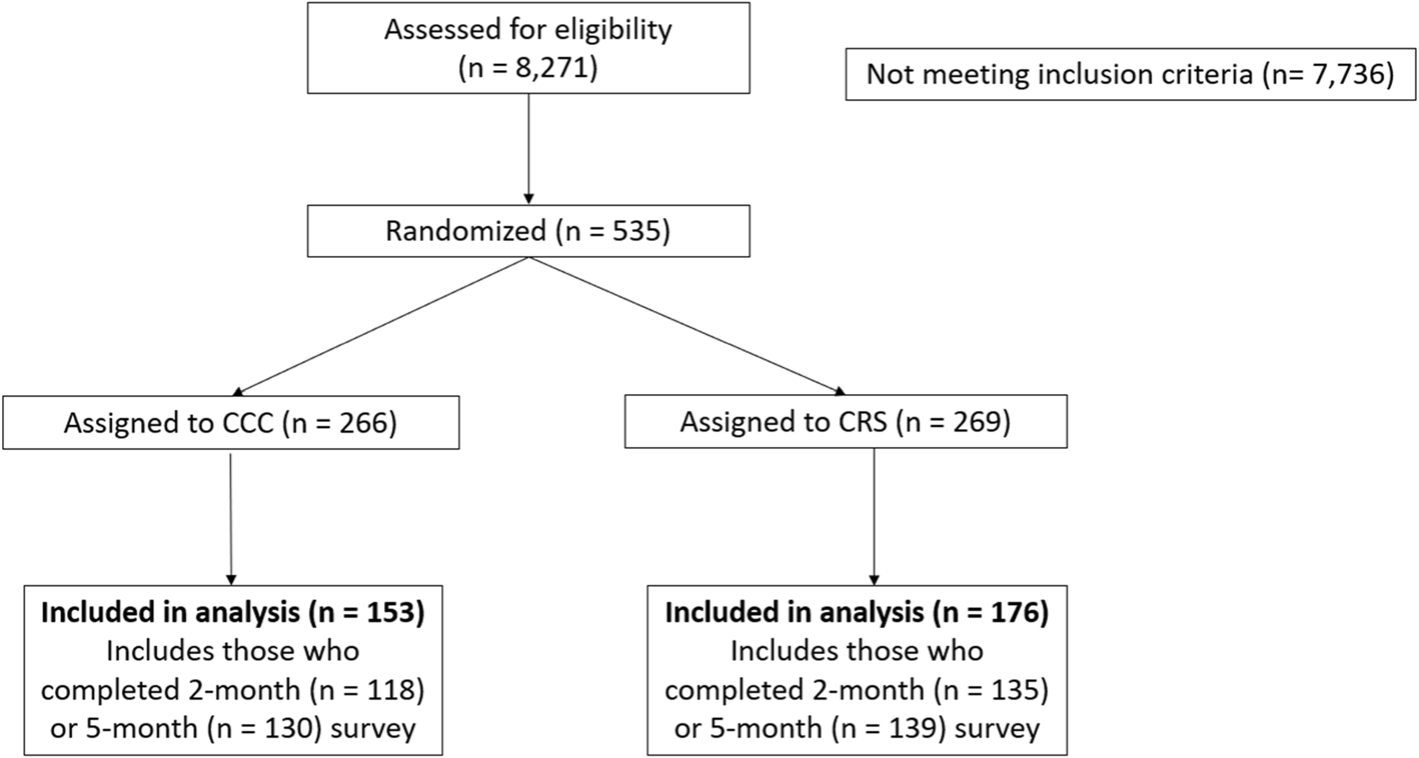
CONSORT flow diagram for randomized participants

During the enrollment period, 80% of patients who were eligible across both clinics completed the SHQ and 535 were ultimately randomized (Fig. 1): 269 to CCC and 266 to CRS.

Three hundred and twenty-nine (61%) completed at least one follow-up survey and were included in analyses (Table 1). About two-thirds of the sample were female and 40% were over the age of 60 and more than half (58%) identified as White. Almost two-thirds of participants fell into a moderate or higher morbidity ACG category. The majority of participants in this study were from Clinic A (71%) which serves about three times the number of patients at Clinic B.

**Table 1:** Participant characteristics

Characteristics	CCC (*n* = 153)	CRS (*n* = 176)	Total (*n* = 329)	Standardized mean differences
Administrative sex, n(%)				0.016
Female	102 (66.7%)	118 (67.0%)	220 (66.9%)	
Male	51 (33.3%)	58 (33.0%)	109 (33.1%)	
Age, n(%)				0.021
< 18	9 (5.9%)	14 (8.0%)	23 (7.0%)	
18–40	41 (26.8%)	50 (28.4%)	91 (27.7%)	
41–60	38 (24.8%)	46 (26.1%)	84 (25.5%)	
60 +	65 (42.5%)	66 (37.5%)	131 (39.8%)	
Administrative Race and ethnicity, n(%)				0.214
African American or Black	9 (5.9%)	8 (4.5%)	17 (5.2%)	
Native American or Alaska Native	0 (0%)	1 (0.6%)	1 (0.3%)	
Asian	3 (2.0%)	5 (2.8%)	8 (2.4%)	
Hispanic	6 (3.9%)	13 (7.4%)	19 (5.8%)	
Multiracial	6 (3.9%)	9 (5.1%)	15 (4.6%)	
Native Hawaiian or Pacific Islander	3 (2.0%)	3 (1.7%)	6 (1.8%)	
White	100 (65.4%)	92 (52.2%)	192 (58.4%)	
Other	3 (2.0%)	5 (2.8%)	8 (2.4%)	
Unknown	23 (15.0%)	40 (22.7%)	63 (19.1%)	
Insurance type, n(%)				0.076
Commercial	51 (33.3%)	71 (40.3%)	122 (37.1%)	
Individual	5 (3.3%)	5 (2.8%)	10 (3.0%)	
Medicaid	21 (13.7%)	26 (14.8%)	47 (14.3%)	
Medicare	67 (43.8%)	56 (31.8%)	123 (37.4%)	
No coverage	9 (5.9%)	18 (10.2%)	27 (8.2%)	
Adjusted clinical group utilization bands, n(%)				0.196
Non-user	2 (1.3%)	6 (3.4%)	8 (2.4%)	
Healthy users	5 (3.3%)	6 (3.4%)	11 (3.3%)	
Low	3 (2.0%)	10 (5.7%)	13 (4.0%)	
Moderate	53 (34.6%)	57 (32.4%)	110 (33.4%)	
High	34 (22.2%)	27 (15.3%)	61 (18.5%)	
Very High	24 (15.7%)	20 (11.4%)	44 (13.4%)	
Missing	32 (20.9%)	50 (28.4%)	82 (24.9%)	
Binary adjusted clinical group utilization bands, n(%)				0.118
No user/Low/Healthy	10 (6.5%)	22 (12.5%)	32 (9.7%)	
Moderate/High/Very High	111 (72.5%)	104 (59.1%)	215 (65.3%)	
Missing	32 (20.9%)	50 (28.4%)	82 (24.9%)	
Clinic				0.01
A	109 (71.2%)	126 (71.6%)	235 (71.4%)	
B	44 (28.8%)	50 (28.4%)	94 (28.6%)	
Baseline count of needs, mean (SD)	1.63 (1.25)	1.78 (1.23)	1.71 (1.24)	

Participant demographics were mostly balanced between programs, except for race and ethnicity. There were more participants who identified as Hispanic in CRS (7.4%) than CCC (3.9%) and a smaller proportion of White participants in CRS (52.2%) compared to CCC (65.4%). We observed that more CRS participants had a “Low” ACG level compared to CCC (2.0 and 5.7%, respectively). Fewer CRS participants also fell into a “High” ACG level relative to CCC (15.3% and 22.2%, respectively).

### Descriptive analyses

**Fig. 2:**
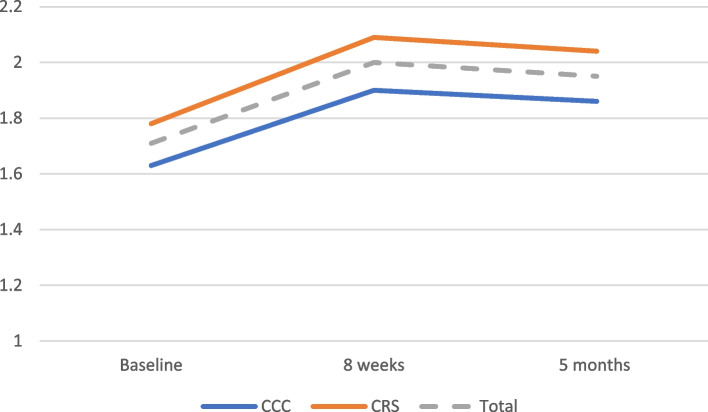
Change in total count of needs over time, by program

The unadjusted baseline mean count of needs for CCC participants was 1.63 (SD = 1.25) (Fig. 2). This increased to 1.90 (SD = 1.98) at 2 months and slightly fell to 1.86 (SD = 1.90) at 5 months. Among CRS participants, the mean count of needs at baseline was 1.78 (SD = 1.23) and this was not significantly different from CCC. Participants in CRS reported 2.09 (SD = 2.12) needs at 2 months followed by 2.04 (SD = 1.98) needs at 5 months.

Financial strain, utilities assistance, and difficulty paying for medical care, medicine, or medical supplies were the top three needs reported among all participants and across timepoints (Table 2). Specifically, 17% of all participants reported financial strain at baseline, followed by 24% at 2 months and 27% at 5 months. We found that 27% requested utilities assistance at baseline, 24% at 2 months, and 32% at 5 months. Lastly, 35% reported difficulty paying for medical care at baseline and this slightly decreased over time with 33% at 2 months and 31% at 5 months.

**Table 2:** Prevalence of social needs at each timepoint

	**CCC**			**CRS**			**Total**		
	**Baseline (*n* = 153)**	**2 months (*n* = 118)**	**5 months (*n* = 130)**	**Baseline (*n* = 176)**	**2 months (*n* = 135)**	**5 months (*n* = 139)**	**Baseline (*n* = 319)**	**2 months (*n* = 253)**	**5 months (*n* = 269)**
**Need**									
Food insecurity	36 (23.5%)	35 (29.7%)	33 (25.4%)	46 (26.1%)	42 (31.1%)	42 (30.2%)	82 (24.9%)	77 (30.4%)	75 (27.9%)
Housing instability	18 (11.8%)	21 (17.8%)	18 (13.8%)	36 (20.5%)	27 (20.0%)	27 (19.4%)	54 (16.4%)	48 (19.0%)	45 (16.7%)
Utilities assistance	30 (19.6%)	41 (34.7%)	35 (26.9%)	58 (33.0%)	42 (31.1%)	50 (36.0%)	88 (26.7%)	83 (32.8%)	85 (31.6%)
Financial strain	24 (15.7%)	28 (23.7%)	37 (28.5%)	32 (18.2%)	32 (23.7%)	36 (25.9%)	56 (17.0%)	60 (23.7%)	73 (27.1%)
Transportation issues	27 (17.6%)	16 (13.6%)	18 (13.8%)	21 (11.9%)	20 (14.8%)	24 (17.3%)	48 (14.6%)	36 (14.2%)	42 (15.6%)
Caregiving	14 (9.2%)	11 (9.3%)	14 (10.8%)	13 (7.4%)	16 (11.9%)	12 (8.6%)	27 (8.2%)	27 (10.7%)	26 (9.7%)
Loneliness or social isolation	23 (15.0%)	18 (15.3%)	24 (18.5%)	29 (16.5%)	29 (21.5%)	27 (19.4%)	52 (15.8%)	47 (18.6%)	51 (19.0%)
Employment	11 (7.2%)	12 (10.2%)	19 (14.6%)	14 (8.0%)	18 (13.3%)	11 (7.9%)	25 (7.6%)	30 (11.9%)	30 (11.2%)
Childcare	7 (4.6%)	5 (4.2%)	6 (4.6%)	10 (5.7%)	10 (7.4%)	9 (6.5%)	17 (5.2%)	15 (5.9%)	15 (5.6%)
Paying for medical care, medicine, or medicine supplies	60 (39.2%)	37 (31.4%)	38 (29.2%)	55 (31.3%)	46 (34.1%)	45 (32.4%)	115 (35.0%)	83 (32.8%)	83 (30.9%)
Do not want help		28 (23.7%)	35 (26.9%)		37 (27.4%)	36 (25.9%)		65 (25.7%)	71 (26.4%)

### Intent-to-treat regression analyses

Unadjusted results showed no statistically significant differences between programs at both 2- and 5-month follow-up timepoints (Table 3). We found that CRS participants reported 0.23 (95% CI: −0.725, 1.183) more needs at 2 months and 0.65 (CI: −0.233, 1.540) more needs at 5 months, relative to CCC participants. Our adjusted analysis results were also not statistically significant. We found that CRS had 0.08 (95% CI: −0.710, 0.864) more needs at 2 months and 0.42 (CI: −0.288, 1.126) more needs at 5 months compared to CCC. Our findings from the sensitivity analysis to account for selection bias were consistent with these results (Appendix).

**Table 3:** Unadjusted and adjusted model results

Variable	Mean count of needs	95% CI		RR	RR 95% CI		*p*-value	Joint test *p*-value
***Unadjusted results***								
2 months								0.343
CCC	5.34	4.634	6.037					
CRS	5.56	4.950	6.178	1.04	0.860	1.226	0.639	
Difference	0.23	-0.725	1.183					
5 months								
CCC	4.62	4.017	5.229					
CRS	5.28	4.667	5.886	1.14	0.936	1.347	0.150	
Difference	0.65	-0.233	1.540					
***Adjusted results***								
2 months								0.507
CCC	4.41	3.229	5.583					
CRS	4.48	3.232	5.734	1.02	0.838	1.197	0.847	
Difference	0.08	-0.710	0.864					
5 months								
CCC	3.76	2.592	4.923					
CRS	4.18	2.939	5.415	1.11	0.914	1.310	0.244	
Difference	0.42	-0.288	1.126					

### As-treated analysis

Unadjusted findings showed a statistically significant relationship between receipt of resources from CRS and time (*p* = 0.001). Specifically, we found that those who received CRS resources had 0.91 fewer needs (95% CI: −1.975, 0.161) at 2 months and a significant difference at 5 months with those who received CRS reporting 2.38 fewer needs (95% CI: −3.432, −1.324) than those who did not.

## Discussion

Our study aimed to examine the effects of primary care-based social health integration on social needs by comparing two social health support programs over time. This study advances the social health literature by documenting the impact of one of the first social health integration programs in a primary care setting to address a broad set of social needs among a general patient population. We also reported the prevalence of social needs at each time point as well as patient-level outcomes to understand the effects on needs resolution over time.

Our findings from the primary intent-to-treat analysis showed that those assigned to CRS, a local and clinic-based program, did not significantly change the number of social needs that participants reported compared to those assigned to CCC, a national-level centralized call center that provides participants with resource information. These findings remained consistent after accounting for potential selection bias. However, our as-treated analysis results indicate that those who received support from CRS had a lower count of social needs over time than those who did not.

There are several factors to consider when interpreting these findings. For our primary intent-to-treat analysis, we compared CRS to an active comparison group of CCC which was modified from a participant-activated program to one in which CCC staff-initiated contact. Additionally, CRS worked on-site and received warm handoffs prior to the pandemic. During the evaluation, nearly all CRS contacts occurred virtually and after participants were randomized which means these initial contacts occurred after their primary care appointment. This created more similarity between CRS and CCC, contributing towards greater difficulty in distinguishing the effects between programs over time. We observed similar counts of needs across both programs and did not find significant differences over time.

Higher counts of social needs at follow-up compared to baseline may reflect an increase in participants’ trust in the healthcare system, leading to higher comfort in reporting social needs. It is also important to note that the timing of the 2-month follow-up survey window fell during winter 2022. Individuals faced higher prices for utilities and other goods, and there was an increased risk of economic downturn during this time [35, 36]. This may have been reflected in the increase of social needs at 2 months in the entire sample.

There is also no consistent method to measure social needs resolution or timeline during which we can expect to observe resolution. We measured resolution by participants’ self-report data. It may be more beneficial to use successful resource connection as a measure rather than the presence of social needs. This would require investment in social service organizations to ensure they have the ability and capacity to support social needs [37, 38]. There would also need to be greater communication and data management capacity to ensure that information is shared between healthcare systems and organizations. Lastly, social health resources and referrals for patients often temporarily address social needs, rather than provide systemic or structural solutions to social needs [39]. The cyclical nature of social needs is reflective of these short-term solutions.

Additionally, our as-treated findings highlighted the importance of communication and follow-up between social health support staff and participants to ensure adequate assistance was provided. However, it is possible that those who received resources from CRS may already be more engaged with the healthcare system, giving them greater ability to seek out assistance and pursue resources to address their social health needs. An encounter with CRS or receipt of resources does not guarantee that participants actively used those resources or that participants’ needs were ultimately addressed.

There were a few limitations to this study. First, there may be concerns about the generalizability of our findings to other settings. However, recent literature suggests that characteristics of patients attributed to KP clinics are generally reflective of their communities [40]. Our results are also from a primary care-based program and this setting often serves more generalized patient populations. Second, our analyses compared CRS to an active comparison group due to external factors, minimizing potential differences between CRS and a true control group. Third, patients were only eligible for randomization if they had a primary care appointment scheduled during the enrollment period, meaning that patients had to already be engaged with the healthcare system. It is possible that patients with complex needs, and who could also benefit from these programs, are not interacting with their primary care providers. Lastly, it was not possible to measure patients’ engagement with the resources they received through CRS, CCC, or from elsewhere. Patients’ successful connection with resources could serve as an alternative outcome measure of social health integration programs, and serves as a future area of research.

## Conclusions

This study provided an example of social health universal screening and subsequent connection to a social health support program in a primary care setting. Health systems will continue to adopt social health integration initiatives, particularly in response to new policy and reporting requirements. It is essential to understand the effectiveness of these types of programs in real-world settings so that health systems can identify appropriate resources and strategies. While we did not observe significant differences in our intent-to-treat analysis between social health support programs over time, our as-treated analysis showed statistically significant and meaningful differences when we assessed the effect of receiving CRS support.

Future research should continue to build upon this study by strengthening intervention and evaluation components in order to identify best practices and their associated effectiveness. For example, a distinctly different or true comparison group would allow researchers to determine if certain program components work well for reducing social needs and provide justification for health systems to invest in necessary resources. While there is no standard measure of social needs resolution, our definition focused on the change in count of needs over time and did not guarantee participants’ successful resource connection or interaction with a CRS or CCC agent. Examining these process measures or focusing on successful social service connection may be more relevant to better understand these programs. Additionally, a larger sample size for as-treated analyses would be extremely beneficial to assess the effects of full engagement with social health support programs on social needs.

## Supplementary Information

Additional file 1. Appendix file that includes additional information about the universal screener and figure of the final SHQ-9, a description of the Heckman selection model to address selection bias, and details about the development of the sample for the as-treated analysis.

## Data Availability

The dataset generated and used for this study is not publicly available but is available from the corresponding author on reasonable request.

## Abbreviations

ACG: Adjusted Clinical Group
EHR: Electronic health record
CCC: Connections Call Center
CRS: Community Resource Specialist
KP: Kaiser Permanente
KPWA: Kaiser Permanente Washington
SHQ: Social Health Questionnaire
SMD: Standardized mean difference
