# Supplementary material for: Comparison of clinic-based assistance versus a centralized call center on patient-reported social needs: findings from a randomized pilot social health integration program
Source: BMC Public Health. 2025 Mar 28;25:1171. doi: 10.1186/s12889-025-22334-x (PMC11951525; doi:10.1186/s12889-025-22334-x)
Supplement: Supplementary file 1 — Additional file 1. Appendix file that includes additional information about the universal screener and figure of the final SHQ-9, a description of the Heckman selection model to address selection bias, and details about the development of the sample for the as-treated analysis. [file 12889_2025_22334_MOESM1_ESM.docx]

**Title**

Comparison of clinic-based assistance versus a centralized call center on patient-reported social needs: Findings from a randomized pilot social health integration program

**Authors**

Ammarah Mahmud^1,2^, Meagan C. Brown, Edwin S. Wong, India J. Ornelas, Robert Wellman, Roy Pardee, Sophia Mun, Ariel Singer, Emily Westbrook, Kathleen Barnes, Heidi Den Haan, Cara C. Lewis

 1. Department of Health Systems and Population Health, University of Washington, Seattle, WA.

2. Kaiser Permanente Washington Health Research Institute, Seattle, WA.

**APPENDIX**

**Universal Screener**

Patients received a Social Health Questionnaire (SHQ-9) during their check-in for primary care appointments (Appendix Figure 1). The first 8 items ask patients about four social risks: financial strain, food insecurity, transportation issues, and housing insecurity. The final item asks patients if they would like to receive assistance for any of the 10 social needs listed. This screener is unique to the Washington Region of Kaiser Permanente (KPWA) and the items were derived from two sources. First, items were selected to align with Epic’s established Social Determinants of Health (SDOH) Wheel for the social risk domains. The Washington Region had previously used the “Your Current Life Situation” (YCLS) survey which asked patients about their social and economic needs The YCLS has been psychometrically tested among Accountable Communities of Health patients and the item asking specifically about social needs was pulled from the YCLS.^2^

**Appendix Figure 1: Social Health Questionnaire**

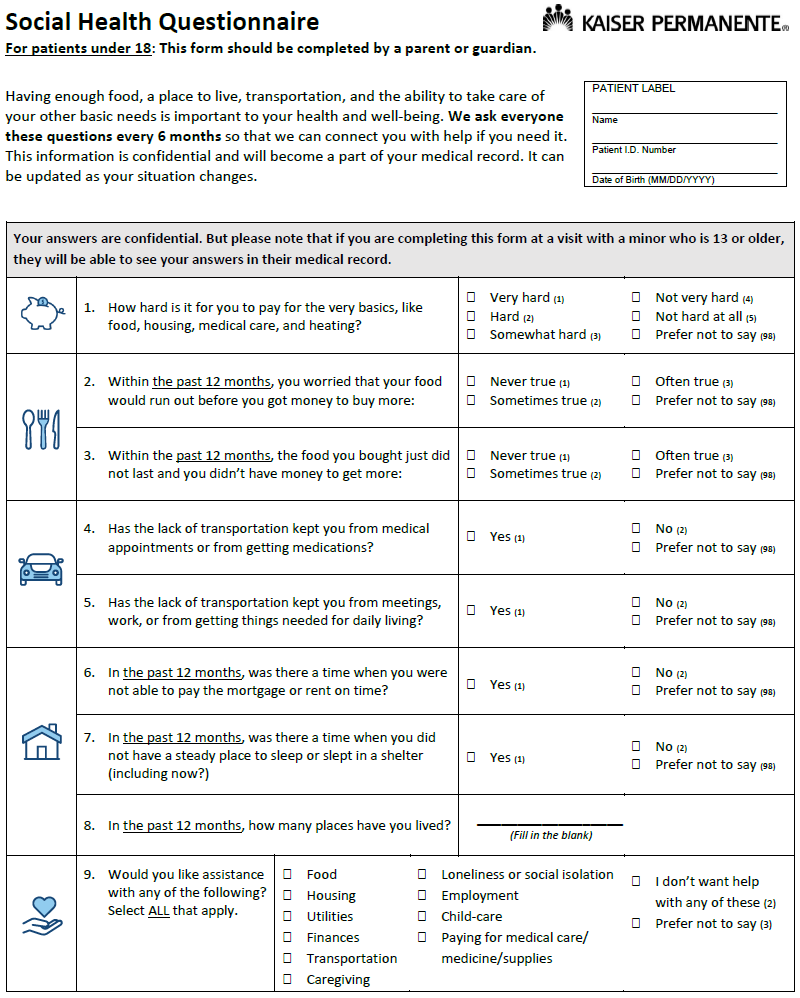


**Heckman selection model to address selection bias**

We conducted a sensitivity analysis using our intent-to-treat sample to address potential selection bias as participants were only included in the primary analysis analytic sample if they completed at least one follow-up survey. We specifically used the “heckpoisson” command in Stata which consists of a two-part model. The first part consists of the selection model in which the dependent variable is a binary variable indicating if the participant completed any survey and therefore in the analytic sample. The second part is a Poisson outcome model in which the dependent variable is the count of needs at follow-up.

We included the same set of independent variables in both parts of the model which included program, time (2-month; 5-month), sex, age, clinic, comorbidity as measured by the Adjusted Clinical Group (ACG) algorithm, race and ethnicity, and count of needs at baseline. We also added binary variables on the presence of the following individual needs at baseline: finances, utilities, employment, caregiving, and difficulty paying for medical care, medicine, or supplies. We did not include the five other individual needs reported at baseline because of issues with model convergence. An interaction term between program and time was included in the outcome model only.

We estimated the marginal effects between groups at each timepoint and found that those in CRS reported 0.05 (95% CI: -0.567, 0.469) fewer needs at 2 months and 0.28 (95% CI: -0.227, 0.782) higher needs at 5 months compared to CCC.

**Sample for as-treated analysis**

We conducted an as-treated analysis to measure the effect of receiving CRS relative to those who were assigned to CRS but did not receive support. Appendix Figure 2 shows the randomization scheme of this evaluation. The top row shows possible universal screener responses. Grey boxes indicate that patients who have a social risk but do not desire assistance, and were excluded from the study. The red box includes the analytic sample of the secondary as-treated analysis. We defined receipt of CRS based on levels on engagement and this analysis acknowledged that adherence to CRS could vary after assignment.

**Appendix Figure 2: Analytic sample of as-treated analysis**
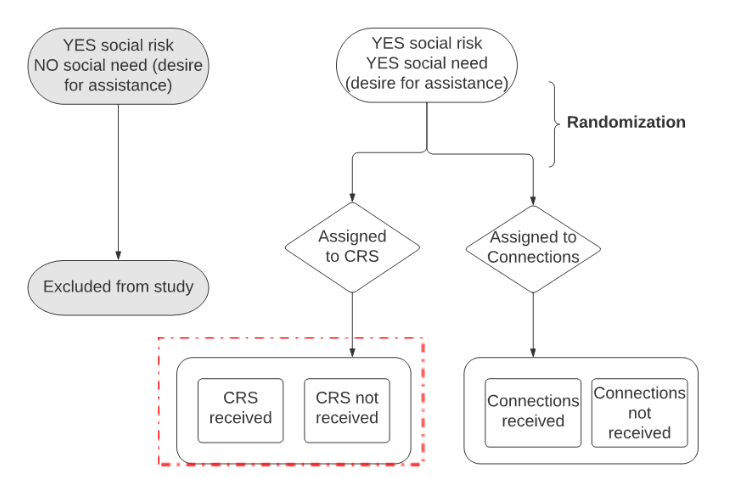


To identify who received to CRS, the evaluation team reviewed CRS case notes to categorize patients into one of four levels of engagement: (1) Full, if they had a conversation with the agent and received resource information; (2) Ineligible, if they spoke to an agent and received a referral but were ineligible for the service; (3) Partial, if they had a conversation with an agent but did not receive a referral or refused assistance; and (4) None, if there was no response or they immediately hung up on the agent. Two members from the research team reviewed either CRS or Connections Call Center (CCC) case notes. If the case note did not explicitly fall into an engagement category, a subset of the evaluation team made the final decision. We dichotomized CRS engagement and classified those who fell in the “Full” and “Ineligible” categories as patients who received CRS. We also included data from 1 patient who was assigned to CCC but received CRS.

Receipt of CRS can be dependent on participants’ ability and motivation to resolve their social needs. For example, many case notes documented participants’ refusal to engage with the CRS or their interest in receiving resources but inability to pursue them at the time. This may have contributed to selection bias in that those who received CRS had to engage with CRS and accept resource information. To address this and support our ability to estimate a causal effect, we used propensity score weights to create a balanced sample.

We developed propensity score weights at baseline using the “twang” package in R to balance groups who received and did not receive CRS support using observable characteristics.^3^ This package applies machine learning models that enable the data-driven inclusion of non-linearities and interactions of covariates into the propensity score model. In this manner, the propensity score approach in the twang package is able to quickly balance the distribution of variables, regardless of the number and types of covariates.^4^ Diagnostic tools under this package are helpful for understanding the quality of the final estimates.

The covariates in the propensity score model are displayed in Appendix Table 1. We used a combination of individual, neighborhood, and clinic-level characteristics hypothesized to influence receipt of CRS. Individual-level characteristics included demographic information as well as diagnoses for 26 chronic conditions.

**Appendix Table 1: Propensity score model covariates**

| **Variable** | **Description** |
| --- | --- |
| *Individual level* |  |
| Adjusted clinical group (ACG) | Morbidity classification collapsed into binary resource utilization bands: Non-User/Healthy/Low and Moderate/High/Very High at baseline and follow-up |
| Race and ethnicity | Administrative data aggregated into 5 categories: African American/Black; Hispanic; Multiracial; Other; Unknown |
| Type of insurance | Type of insurance: Commercial, Individual, Medicaid, Medicare, or No coverage |
| Congestive heart failure | Yes/No disease flag |
| Valvular disease | Yes/No disease flag |
| Peripheral vascular disease | Yes/No disease flag |
| Depression | Yes/No disease flag |
| Paralysis | Yes/No disease flag |
| Other neurological disorders | Yes/No disease flag |
| Chronic pulmonary disease | Yes/No disease flag |
| Diabetes | Yes/No disease flag |
| Diabetes with chronic complications | Yes/No disease flag |
| Hypothyroidism | Yes/No disease flag |
| Renal failure | Yes/No disease flag |
| Liver disease | Yes/No disease flag |
| Peptic ulcer disease | Yes/No disease flag |
| AIDS | Yes/No disease flag |
| Metastatic cancer | Yes/No disease flag |
| Tumor | Yes/No disease flag |
| Rheumatoid arthritis/collagen vascular disease | Yes/No disease flag |
| Coagulopathy | Yes/No disease flag |
| Obesity | Yes/No disease flag |
| Weight loss | Yes/No disease flag |
| Chronic blood loss anemia | Yes/No disease flag |
| Deficiency anemia | Yes/No disease flag |
| Alcohol abuse | Yes/No disease flag |
| Drug abuse | Yes/No disease flag |
| Psychoses | Yes/No disease flag |
| Hypertension | Yes/No disease flag |
| *Neighborhood level* | |
| Neighborhood deprivation index | Neighborhood deprivation index at census tract level in 2020 |
| *Clinic level* | |
| Number of primary care providers | Number of primary care providers at each clinic |
| Number of full time CRS | Number of full time equivalent CRSs affiliated with each clinic |

We used predicted values from the propensity score model to estimate average treatment effect in the population (ATE) weights. We also applied an “es.mean” stop method to balance covariates between groups. This method estimates weights by minimizing the standardized absolute mean difference of covariates. Prior to weighting, we observed two variables that were the most imbalanced. This included participants that identified as White and a diagnosis of psychoses. After applying weights, we verified that covariates had a standardized mean difference less than 0.2 (Appendix Figure 3). Boxplots in Appendix Figure 4 illustrated the spread of propensity scores and overlap in the as-treated and comparison groups.

**Appendix Figure 3: Absolute standardized difference of covariates before and after weights**


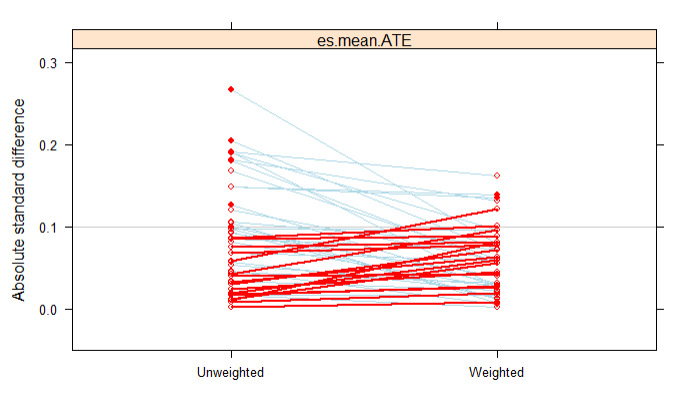


**Appendix Figure 4: Spread of propensity scores in treatment and comparison groups**


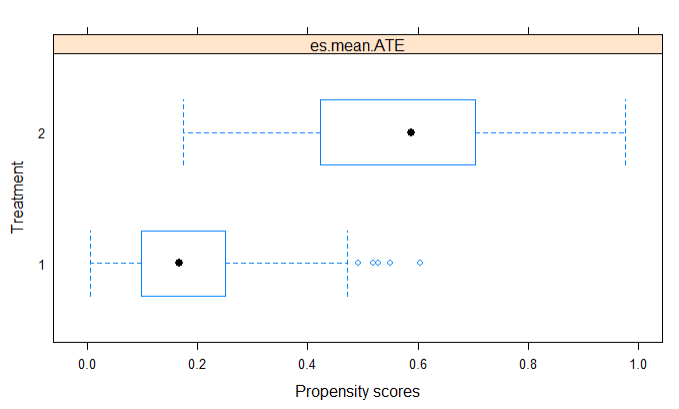


Note: 2 = CRS receipt; 1 = Comparison

We derived propensity score weights from the model and applied them to the data restricted to the analytic sample for the as-treated analysis using the “svyset” command in Stata.^5^ This provided a treatment sample size of 104 participants and 144 in the comparison group. Similar to the primary intent to treat analysis, our outcome model was estimated using generalized mixed effects linear model with a Poisson log link distribution and individual-level random effects.

**References**

1. Kaiser Permanente. Your Current Life Situation Survey. Published 2017. Accessed November 17, 2023. https://sirenetwork.ucsf.edu/tools-resources/resources/your-current-life-situation-survey#:~:text=Kaiser%20Permanente%27s%20Your%20Current%20Life,%2C%20stress%2C%20and%20social%20isolation.

2. Lewis CC, Wellman, R, Jones SMW, et al. Comparing the performance of two social risk screening tools in a vulnerable subpopulation. *J Family Med Prim Care*. 2020;9(9):5926-5034. doi:10.4103/jfmpc.jfmpc_650_20

3. Ridgeway G, McCaffrey DF, Morral AR, Burgette LF, Griffin BA. *Toolkit for Weighting and Analysis of Nonequivalent Groups: A Tutorial for the R TWANG Package*. RAND Corporation; 2014. doi:10.7249/TL136.1

4. McCaffrey DF, Ridgeway G, Morral AR. Propensity Score Estimation With Boosted Regression for Evaluating Causal Effects in Observational Studies. *Psychological Methods*. 2004;9(4):403-425. doi:https://doi.org/10.1037/1082-989X.9.4.403

5. Stata. Svyset - Declare survey design for dataset. Accessed November 17, 2023. https://www.stata.com/manuals/svysvyset.pdf
